# Comparison of end-tidal carbon dioxide and arterial blood bicarbonate levels in patients with metabolic acidosis referred to emergency medicine

**DOI:** 10.15171/jcvtr.2016.21

**Published:** 2016-09-30

**Authors:** Ali Taghizadieh, Mahboub Pouraghaei, Payman Moharamzadeh, Alireza Ala, Farzad Rahmani, Karim Basiri Sofiani

**Affiliations:** ^1^Tuberculosis and Lung Disease Research Center, Tabriz University of Medical Sciences, Tabriz, Iran; ^2^Emergency Medicine Research Team, Tabriz University of Medical Sciences, Tabriz, Iran; ^3^Student’s Research Committee, Tabriz University of Medical Sciences, Tabriz, Iran

**Keywords:** Metabolic Acidosis, Capnography, Blood Gas

## Abstract

***Introduction:*** The routine and gold standard method to diagnose of acid – base disturbance is arterial blood gas (ABG) sampling. Capnography could be used to measure the end-tidal carbon dioxide (ETCO2) levels and ETco2 has a close correlation with the PaCo2. The aim of this study was comparison the ETco2 and arterial blood bicarbonate levels in patients with metabolic acidosis.

***Methods:*** In a descriptive-analytical study that performed in Emergency Department of Emam Reza Medical Research and Training Hospital of Tabriz on patients with metabolic acidosis, ETco2 level and blood bicarbonate levels in 262 patients were evaluated.

***Results:*** Mean of ETco2 and Hco3 levels in patients with metabolic acidosis were 22.29 ± 4.15 and 12.78 ± 3.83, respectively. In all patients, the significant direct linear relationship was found between ETco2 with Hco3 (r = 0.553, *P * < 0.001). We had 4 groups of patients with metabolic acidosis, also there is a significant direct linear relationship between the ETCo2 and the Hco3 level of arterial blood in patients with renal failure (*P * < 0.001 and r = 0.551), sepsis (*P * < 0.001 and r = 0.431), drug toxicity (*P * < 0.001 and r = 0.856), and ketoacidosis (DKA) (*P * < 0.001 and r = 0.559).

***Conclusion: ***According to the results of this study, capnography can be used for primary diagnosis of metabolic acidosis in spontaneously breathing patients who referred to the emergency wards, however, the ABG must be considered as the gold standard tool for diagnosis and guiding the treatment.

## Introduction

The natural balance of acid-base is essential for proper function of cells. This balance is established by the lung, kidney, and serum physiologic buffers. The blood pH is determined by the ratio between the serum bicarbonate concentration and the PaCO2. The metabolic acidosis is a common problem in the emergency department (ED). They are usually hospitalized with the symptoms such as severe vomiting, diarrhea, weakness and lethargy, respiratory distress, renal failure, and the life-threatening acid-base disorders are also observed in some cases. Diagnosis and appropriate treatment of the acid-base disorder could be save the patient’s life.^1^

The arterial blood gas (ABG) is used to evaluate the acid-base status and ABG sampling is an invasive and painful procedure for the patient, and this could lead to a delay in starting treatment by a physician. This method sometimes causes the arterial wall spasm in some cases.^2^ In physiological conditions, the body increases the minute ventilation to compensate for the metabolic acidosis, thus PaCO2 levels would be decreased.^3-5^ Wave capnography in a noninvasive tool and indicate the amounts of expiratory CO2.^6,7^ The Capnography provides the accurate information in terms of the ventilation, perfusion and metabolic status from one breath to the next.^6,8^ If the patient has a normal respiratory function, the difference between ETCO2 and PaCO2 was 2-5 mm Hg, and the amount of Paco2 was greater than the ETCO2. These differences are caused by dead space in the respiratory system that does not participate in breathing.^6^

In a study conducted by Kartal and colleagues, they suggested that the ETCO2 level could demonstrate the severity of metabolic acidosis and mortality in these patients.^2^ Fearon and Steele concluded that in patients with diabetic ketoacidosis (DKA), patients with ETCO2 values less than 29 suffered from DKA and the patients with ETCO2 values more than 36 did not have DKA.^3^ Soleimanpour et al had concluded that capnography is an appropriate tool to rule out the DKA in ED.^9^

Whereas the ABG sampling is an invasive and painful procedure for the patient which is also an expensive method and time-consuming for the medical staff, thus in this research we decided to investigate the capnography as a non-invasive method to diagnose the metabolic acidosis in suspected patients in the ED.

## Materials and Methods

In the descriptive-analytic study that was conducted in the ED of Imam Reza Medical Research and Training Hospital, Tabriz, East Azerbaijan, Iran, 110 000 admission per year, during a year period (Jan 2014–Dec 2014) on the patients with metabolic acidosis referred to the ED, relationship of the ETCO2 values with arterial bicarbonate were evaluated. Imam Reza Medical Research and Training Hospital is a referral governmental general hospital. To determine the sample size, we used sample size formula based on the correlation coefficient of Kartal et al study^2^ (r=0.5 between the value of ETCO2 and HCO3), with confidence coefficient of 95% and α = 0.05, and 210 patients were determined as sample of study, and finally we included 262 patients in our study. The sample size formula was n= (Z_1-α/2_+Z_1-β_)^2^/(ώ)^2^. Inclusion criteria were all patients with suspected sepsis, acute gastroenteritis, renal failure, DKA and other disorders with metabolic acidosis. Exclusion criteria were the chronic obstructive pulmonary disease (COPD) and other respiratory diseases, loss of consciousness, lack of consent to participate in research, the patients who had the mixed disorders, and intolerance the capnography.

At the first time of arrival the patient, after recording the vital signs and the history and physical exam, obtaining informed written consent were taken. If the patient met the inclusion/exclusion criteria, at the same time we have done capnography and ABG sampling. If the ABG result was metabolic acidosis, the patient was included in our study. Capnometry was initially performed for patients by use of the Capnography device (model number: 7100, RESPIRONICS, California Inc, California, USA) in the ED and the ETCO2 values were recorded. The level of arterial pH, PaCO2 and bicarbonate were simultaneously measured based on the ABG results, by use of the AVL 995 ABG analyzer devices and then the results were recorded. Capnography at the same time that ABG samples were taken, were done. To increase the accuracy of capnography results, it was performed for 1 minute. The blood bicarbonate levels lower than 15 meq/L is considered as the acidosis.

The collected data were analyzed by SPSS 17.0.1 (SPSS Inc, Chicago) software. The normal distribution of data was investigated with the Kolmogorov-Smirnov test. Descriptive tests (mean ± SD, prevalence, and percent) were used to describe the data. Independent sample’s Kruskal-Wallis test was used to compare the quantitative data. Receiver operating characteristic (ROC) curve analysis was used to determine the cut-off-point, sensitivity, and specificity of ETCO2 in the diagnosis of metabolic acidosis in various diseases. *P* value ˂ 0.05 was considered statistically significant.

## Results

**Figure 1:**
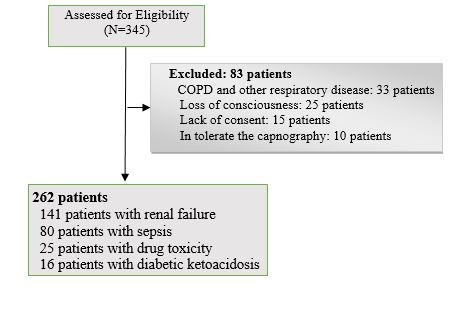
Patients flowchart

In our study, 262 patients with metabolic acidosis were evaluated. The mean±SD age of them was 60.18±19.02 years. About our patients, 143 patients (54.6%) were male and 119 patients (45.4%) were female. 141 patients had renal failure, 80 patients had sepsis, 16 patients had drug toxicity, and others (25 patients) had DKA. Figure 1 shows the patients flowchart of study.

**Figure 2:**
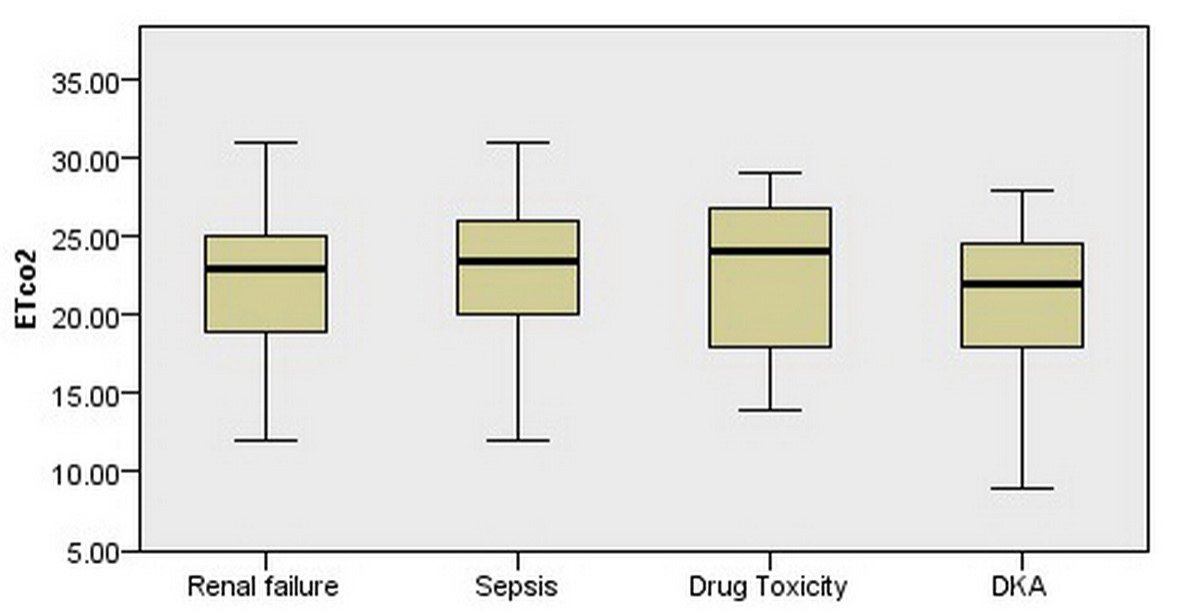
ETCO2 level distribution of patient.

Table 1 shows the demographic features and clinical and laboratory findings of patients based on their disease. Figure 2 shows the ETCO2 level distribution of patient based on their disease. The ETCO2 level in patients is significantly 7.03 ± 5.35 mm Hg less than PaCO2 level. There is a significant indirect linear relationship between the respiratory rate of patients and the ETCO2 level in patients (r = -0.30, *P* < 0.001).

**Table 1:** The vital signs of patients based on their diseases

**Variables**		**Disease**					*** P*** ** value**
		**Renal failure (141 patients)**	**Sepsis (80 patients)**	**Drug toxicity (16 patients)**	**DKA (25 patients)**		
Vital signs	MAP. mm Hg	91.65 ± 22.05	70.57 ± 21.31	88.18 ± 27.29	84.84 ± 25.25	˂0.001
	Heart rate, bpm	90.18 ± 15.75	104.09 ± 20.60	102.13 ± 16.83	106.68 ± 19.19	˂0.001
	Respiratory rate, Per/min	23.04 ± 6.65	23.56 ± 7.74	21.93 ± 5.43	25.80 ± 9.06	0.389
	Body temperature, ^o^C	36.80 ± 59	37.41 ± 0.96	36.69 ± 0.41	36.93 ± 0.73	˂0.001
	O2 sat.	92.53 ± 4.21	91.15 ± 4.89	92.93 ± 3.00	94.08 ± 3.57	0.001
Lab. findings	White blood cell, ×10^9^/L	10394.39 ± 5712.30	12872.13 ± 7897.58	13243.75 ± 7020.92	14176.00 ± 9080.53	0.32
	Hemoglobin, g/L	9.98 ± 2.70	11.49 ± 3.14	13.58 ± 3.85	12.86 ± 3.45	˂0.001
	Urea, mmol/L	170.04 ± 70.97	81.48 ± 50.38	66.50 ± 65.17	93.60 ± 78.18	˂0.001
	Creatinine, mg/dL	8.61 ± 4.94	2.60 ± 2.20	2.10 ± 2.49	2.33 ± 1.79	˂0.001
	Blood sugar, mmol/L	156.90 ± 137.99	187.81 ± 139.92	168.81 ± 127.96	492.16 ± 197.42	˂0.001
ABG results	pH	7.22 ± 0.85	7.25 ± 0.12	7.26 ± 0.16	7.18 ± 0.19	˂0.001
	Hco3, mmol/L	12.54 ± 3.57	13.76 ± 3.62	14.43 ± 3.98	9.99 ± 4.33	˂0.001
	PaCO2, mm Hg	29.61 ± 6.36	29.30 ± 6.08	30.52 ± 6.08	26.66 ± 8.31	0.444
	End tidal CO2, mm Hg	22.09 ± 40.4	22.81 ± 3.78	22.87 ± 4.79	20.76 ± 5.28	0.240

**Figure 3:**
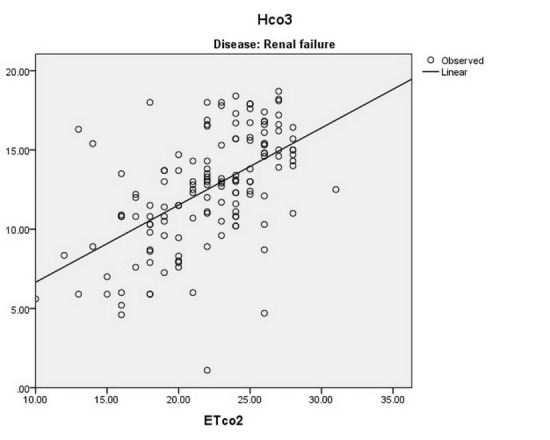
The Correlation between Hco3 and ETCO2 in patients with renal failure

**Figure 4:**
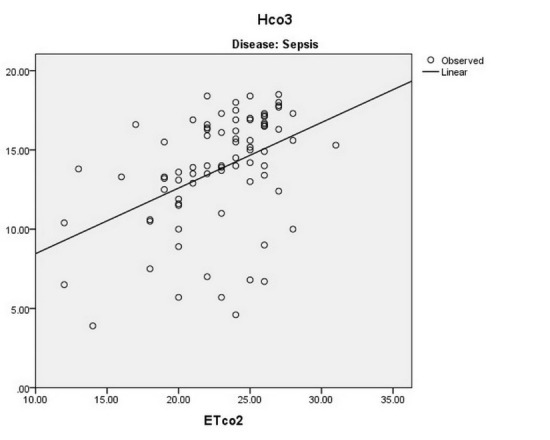
The Correlation between Hco3 and ETCO2 in patients with sepsis.

**Figure 5:**
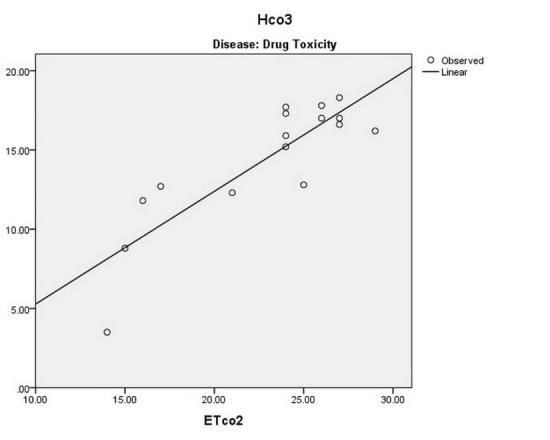
The Correlation between Hco3 and ETCO2 in patients with drug toxicity

**Figure 6:**
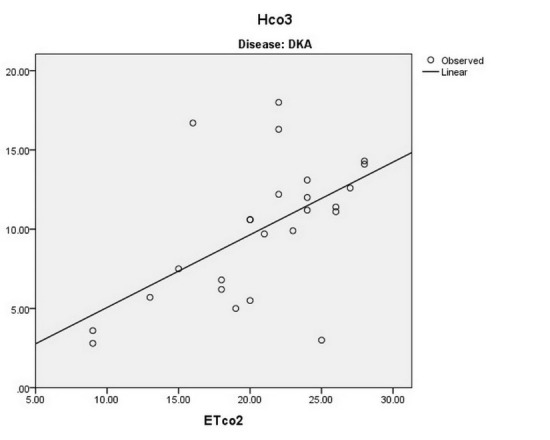
The Correlation between Hco3 and ETCO2 in patients with diabetic ketoacidosis.

There is a significant direct linear relationship between the ETCo2 and the Hco3‏ level of‏ arterial blood in patients with renal failure (*P* < 0.001 and r = 0.551), sepsis (*P* < 0.001 and r = 0.431), drug toxicity (*P* < 0.001 and r = 0.856), and DKA (*P* < 0.001 and r = 0.559) (Figures 3-6).

## Discussion

In our study, 262 patients with metabolic acidosis were evaluated, the ETCO2 and Hco3 level of arterial blood were measured in these patients and compared with each other. There is a statistical significant direct linear relationship between the ETCO2 and the Hco3 level of arterial blood in patients with metabolic acidosis.

The ABG is the common method to determine the presence of acidosis in the patients. The arterial blood sampling is an invasive and painful procedure with several side effects which is also an expensive method and time-consuming for the medical staff thus it seems to be reasonable to find an alternative non-invasive method for determining the presence of the metabolic acidosis.^6,7^

Use of non-invasive methods instead of the ABG has been reviewed in various studies. In the study conducted by Solana García et al on the acute gastroenteritis dehydrated children with metabolic acidosis, following the performance of capnography, the researchers suggested that it could be used as a noninvasive method for measuring metabolic acidosis in dehydrated children with acute diarrhea.^10^ In a study that conducted by Moses and colleagues, they discussed that there was a significant relationship between the amount of PaCO2 in the blood and ETCO2 of the patients.^11^ In a study conducted by Garcia and colleagues, they demonstrated that ETCO2 is directly correlated with the PaCO2 and inversely correlated with respiratory rate.^12^ In our study, similar to the above findings, ETCO2 is indirectly correlated with respiratory rate (*P* < 0.001, r = -0.30).

In a study conducted by Gilhotra and Porter, they demonstrated that there were no patients with ETCO2 level greater than 30 mmHg suffering from DKA.^13^ In a study conducted by Fearon and Steele, they observed that there was a significant linear relationship between the level of ETCO2 and PaCo2 in these cases.^3^ In a study conducted by Kheng and Rahman, they proposed that there was a significant correlation in the ETCO2 of the patients with systolic and diastolic blood pressure and between the HCo3 and base excess in the patients with septic shock.^14^ In a study conducted by McGillicuddy et al, they indicated that the ETCO2 level less than 35 mm Hg is effective in predicting the multiple organ failures in patients.^15^ In a study conducted by Soleimanpour and colleagues, they demonstrated that capnography values greater than 24.5 mm Hg accurately exclude of DKA in patients suspected.^9^

## Limitations

One of the limitations of our study was that we had not control group to compare data and calculate the cut-off point for the ETCO2 level in the diagnosis of metabolic acidosis. Another limitation of our study was that we could not perform capnography in patients with nausea and vomiting.

## Conclusion

Results obtained from this study show that performing capnography is recommended for detection of metabolic acidosis in aware patients suspected to acid-base disorders. According to the results of this study, capnography can be used for primary diagnosis of metabolic acidosis in spontaneously breathing patients who referred to the emergency wards, however, the ABG must be considered as the gold standard tool for diagnosis and guiding the treatment.

## Ethical issues

This study approved by the ethics committee of the Tabriz University of Medical Sciences with number 6273 at 22.10.2013.

## Competing interests

All authors declare no competing financial interests exist.

## Acknowledgments

The authors are grateful to all the health personnel and patients who participated in the study, in addition to the data collectors, supervisors and administrative staff of the ED of Imam Reza Hospital. This article was written based on dataset of Karim Basiri Sofiani’s specialty thesis entitled “Comparison of end tidal carbon dioxide (ETCO2) and arterial blood bicarbonate levels in patients with metabolic acidosis referred to ED”, registered in Tabriz University of Medical Sciences. [Number: 92/3-8/15, Approved date: 04.01.2013].
